# Task shifting in active management of the third stage of labor: a systematic review

**DOI:** 10.1186/s12884-018-1677-5

**Published:** 2018-02-06

**Authors:** Tessa M. Raams, Joyce L. Browne, Verena J. M. M. Festen-Schrier, Kerstin Klipstein-Grobusch, Marcus J. Rijken

**Affiliations:** 1 Julius Global Health, Julius Center for Health Sciences and Primary Care, University Medical Center Utrecht, Heidelberglaan 100, 3584 Utrecht, The Netherlands; 2 Division of Epidemiology & Biostatistics, School of Public Health, Faculty of Health Sciences, University of the Witwatersrand, Johannesburg, South Africa; 3 Department of Obstetrics and Gynecology, University Medical Center Utrecht, Utrecht, The Netherlands

**Keywords:** Active management of the third stage of labor, Community health workers, Low- and middle-income countries, Postpartum hemorrhage, Self-administration, Task shifting, Traditional birth attendants

## Abstract

**Background:**

Active management of the third stage of labor (AMTSL) describes interventions with the common goal to prevent postpartum hemorrhage (PPH). In low- and middle-income countries, implementation of AMTSL is hampered by shortage of skilled birth attendants and a high percentage of home deliveries. Task shifting of specific AMTSL components to unskilled birth attendants or self-administration could be a strategy to increase access to potentially life-saving interventions. This study was designed to evaluate the effect, acceptance and safety of task shifting of specific aspects of AMTSL to unskilled birth attendants.

**Methods:**

A systematic search was conducted in five databases in September 2015 to identify intervention studies of AMTSL implemented by unskilled birth attendants or pregnant women themselves. Quality of studies was evaluated with an adapted Cochrane Collaboration assessment tool.

**Results:**

Of 2469 studies screened, 21 were included. All studies assessed implementation of uterotonics (misoprostol tablets or oxytocin injections), administered by community health workers (CHWs), auxiliary midwives, traditional birth attendants (TBAs) or self-administration at antenatal (home) visits or delivery. Task shifting for none of the other AMTSL components was reported. Task shifting of provision of uterotonics reduced the risk of PPH (RR 0.16 to 1) compared to standard care (13 studies, *n* = 15.197). The correct dose and timing was reported for 83.4 to 99.8% (5 studies, *n* = 6083) and 63 to 100% (9 studies, *n* = 8378) women respectively. Uterotonics were recommended to others by 80 to 99.7% (7 studies, *n* = 6445); 80 to 99.4% (5 studies, *n* = 2677) would use the drug at next delivery. Willingness to pay for uterotonics varied from 54.6 to 100% (7 studies, *n* = 6090).

**Conclusion:**

Task shifting of AMTSL has thus far been evaluated for administration of uterotonics (misoprostol tablets and oxytocin injected by CHWs and auxiliary midwives) and resulted in reduction of PPH, high rates of appropriate use and satisfaction among users.

In order to increase AMTSL coverage in low-staffed health facilities, task shifting of uterine massage or postpartum tonus assessment to unskilled attendants or delivered women could be considered. Task shifting of controlled cord traction is currently not recommended.

**Electronic supplementary material:**

The online version of this article (10.1186/s12884-018-1677-5) contains supplementary material, which is available to authorized users.

## Background

Postpartum hemorrhage (PPH) is the leading cause of maternal mortality worldwide and accounts for 34% of maternal deaths in low- and middle-income countries (LMIC) [1]. PPH is defined as blood loss of 500 ml or more within 24 h after delivery and most frequently occurs during the third or fourth stages of labor, the period from delivery of the infant until placental delivery and 2 h postpartum [2]. Active management of the third stage of labor (AMTSL) describes a set of interventions aimed at the prevention of PPH [3, 4]. AMTSL includes the administration of uterotonics (for example oxytocin or misoprostol) preferably within 1 min after delivery to all women, controlled cord traction (CCT) to stimulate placental delivery, uterine massage to activate uterine contraction and assessment of uterine tonus every 15 min during 2 h postpartum to early identify uterine atony [1, 4–6]. The World Health Organization (WHO) recommends AMTSL to be performed by skilled birth attendants and health workers trained in the management of pregnancy and delivery [4]. WHO recommendations regarding implementation are component-dependent: uterotonics are recommended for all delivered women. CCT is considered optional in settings with skilled birth attendants, continuous uterine massage is not recommended if prophylactic oxytocin is provided and uterine tonus surveillance is recommended for all delivered women [4, 7–9].

Almost 60% of births in low- and middle-income countries occur in rural areas outside health facilities and are assisted by traditional birth attendants or family members not (formally) trained in obstetric care. Despite attempts to increase skilled attendance at (home) deliveries, this remains challenging due to a shortage of skilled birth attendants and significant travel distances in rural areas [10, 11]. As a consequence, the implementation of AMTSL is limited despite its paramount importance, especially in areas with low access to emergency obstetric care [5, 11–13].

In 2012, the WHO published recommendations on task shifting in maternal and newborn health care in an attempt to optimize the potential of the existing health workforce [14]. These recommendations include for example the administration of misoprostol by lay health workers to prevent postpartum hemorrhage, but don’t address all components of AMTSL. The effects of task shifting of individual components of AMTSL have never been reviewed [14].

Of the AMTSL interventions, uterotonics are the most effective in terms of prevention of PPH, although the relative contribution of each of AMTSL components has not been well-studied [4, 15]. Oxytocin is the preferred uterotonic drug and recommended by the WHO if skilled birth attendants are available. However, as oxytocin is thermo-unstable it requires cold-chain handling which increases costs. Additionally it should be injected intramuscularly or intravenously by skilled birth attendants. Misoprostol is considered a safe, cheap and only slightly less effective alternative [16–19]. The WHO recommends the provision of misoprostol by community health workers for PPH prevention in rural areas and homebirths in absence of skilled attendance [4]. Community distribution of misoprostol to pregnant women for self-administration is also widely explored as an option, but currently not recommended [20–22].

The aim of this systematic review is to evaluate the evidence on the effect, women’s acceptance and safety of task shifting of different components of AMTSL to unskilled birth attendants or self-administration.

## Methods

### Protocol

This review was designed in accordance with the PRISMA guidelines [23].

### Eligibility criteria

#### Type of study

Randomized controlled trials (RCTs) or quasi-experimental trials evaluating the effect of implementation of components of AMTSL to unskilled birth attendants or delivering women on the incidence of PPH compared to the current situation, which is mostly no implementation of components of AMTSL at all. No restrictions on language and publication date were applied. Case reports, reviews and proceedings were excluded.

#### Type of participants

Women delivering in a community setting or health facility center in LMIC without skilled birth attendants present. Skilled birth attendants are defined as accredited health professionals (midwife, nurse) who are trained to assist pregnancies and postpartum care. Unskilled birth attendants are nonprofessionals, educated in a specific task of pregnancy care and postpartum care [24].

#### Type of intervention

Trials evaluating the effect on outcome measures of implementation of administration of uterotonics (oxytocin or misoprostol), controlled cord traction, uterine massage, assessment of uterine tonus during 2 hpostpartum to unskilled birth attendants in areas without standardized postpartum care according to AMTSL. By evaluating the effect and safety of AMTSL performed by unskilled birth attendants, effect and safety of task shifting is indirectly measured. Task shifting is defined as a process in which tasks are moved from skilled to less skilled health workers [14].

#### Type of outcome measures

The primary outcome measure was the incidence of postpartum hemorrhage, defined as blood loss of 500 ml or more in the first 24 h postpartum. Secondary outcomes were women’s acceptance and safety. The first defined as recommending the care to friends or family, willingness to receive the same care at their next delivery and willingness to pay for uterotonics at the next delivery. The latter defined as receiving the care at the correct time during third stage of labor and using correct methods as described in the WHO guidelines [4]. Safety also included adverse effects of interventions.

### Information sources

The search was performed in the following electronic databases: The Cochrane Library (Cochrane Database of Systematic Reviews), EMBASE, Global Health Library, POPLINE and PubMed for all publications up to September 2015. Full texts were available for all included studies. In addition, included studies were screened for additional relevant studies through their reference lists and cross-referenced in Web of Science.

### Search

Synonyms for ‘Active Management of the Third Stage of Labor’ and its components, i.e. ‘uterotonics’, ‘cord traction’, ‘uterine massage’ and ‘uterine tonus’ combined with synonyms for ‘task shifting’ and correlating Mesh terms were used as search strategy. The full search strategy including a list of synonyms is available in the Additional file 1.

### Study selection

After removal of duplicates, the title and abstract of retrieved studies were screened by two independent reviewers (TR, VS) using predefined inclusion and exclusion criteria. Any discrepancies between the two reviewers in this process were discussed and full text accessed if necessary for further clarification. If results were published multiple times, data were used only once from the most complete article.

### Data collection process and data items

Data were extracted from each individual article using a standardized extraction form and included: study design, study setting and location, number of delivered women and population characteristics including age, parity and educational level, intervention as part of AMTSL, by whom this was performed and educational level or training received, incidence of PPH and relative risks with corresponding 95% confidence interval, women’s acceptance including willingness to pay for uterotonics, recommendations to friends and family and wish to receive same care or drugs at next delivery, safety including correct dose, correct time and duration of intervention and reported side effects. The extraction was performed by one reviewer (TR) and a second reviewer was available for discussion or clarification processes. Corresponding authors were contacted in case further information was required.

### Quality assessment

Quality of studies was assessed at study level using an adapted version of the Cochrane Collaboration’s tool for assessing risk of bias for systematic reviews of interventions [25]. Studies were scored for detection bias, evaluating blinding of participants for outcome measurements; attrition bias, evaluating completeness and origin of data; reporting bias, evaluating origin of data, definition and assessment of outcome measurements; and possible confounders. Risk of bias was assigned as low, high or unclear risk, according to the quality assessment tool, attached as a Additional file 2. Any disagreements were discussed until consensus was achieved.

### Summary measures

Incidence of PPH was reported in relative risks (RR) and 95% confidence interval (CI). If these were not reported, they were calculated using SPSS v20.0 software [26]. Women’s acceptance and safety were reported in percentages.

### Synthesis of results

Results were narratively described because the heterogeneity of the interventions and settings did not allow for a meaningful meta-analysis.

## Results

### Study selection

**Fig. 1:**
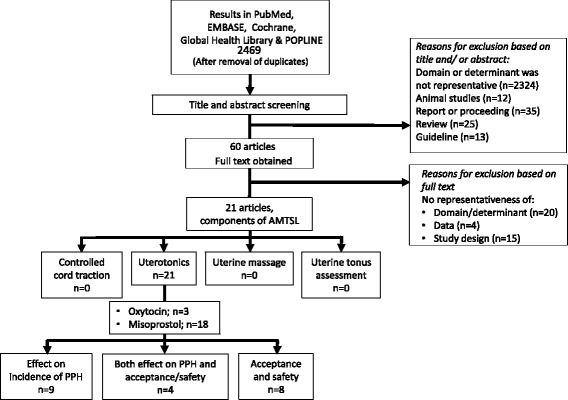
Flowchart of literature search and selection process. PPH: postpartum haemorrhage

The systematic search identified 2469 studies after removal of duplicates, of which 60 studies remained after title and abstract screening [Fig. 1]. Of these studies, 21 met the inclusion criteria. No additional studies were obtained through snowballing. All studies aimed to evaluate effect and safety of implementation of AMTSL to unskilled attendants compared to standard of care (mostly no standardized care). No studies were found reporting on task shifting of components of AMTSL from skilled to unskilled birth attendants.

### Study characteristics

Study characteristics are presented in Tables 1 and 2. Five studies were RCTs [11, 17, 27–29] and sixteen were quasi-experimental trials [5, 10, 12, 13, 22, 30–40]. Studies were published between 2005 and 2014 and reported on 236 to 77.337 included pregnant women in community centers or at home births in LMIC in Africa (48%, *n* = 10) Asia (43%, *n* = 9) and Central America (2%, *n* = 2). Tasks were shifted to community health workers (19%, *n* = 4), auxiliary midwives (19%, n = 4), traditional birth attendants (38%, *n* = 8) or self-administered by delivered women (48%, n = 10).

**Table 1:** Administration of uterotonics. Self-administered, by TBAs, auxiliary midwives or community health workers: RR (95% CI) for PPH (*n* = 13)

Study (first author, year of publication)	Design	Location	Deliveries (N per group)	Population (age, parity, education)^a^	PPH (%)	RR (95% CI)
Misoprostol^b^, self- administered						
Geller et al., 2013 [22]	Quasi exp. trial	30 communities in Ghana	102 (82) misoprostol, 107 (92) control	Age: 26.6 ± 6.7 Parity(2–4): 46% Education: n/a	Misoprostol 1.2% (1/82), Control 3.3% (3/92)	0.38 (0.04–3.57)
Mir et al., 2012 [31]	Quasi exp. trial	Districts of Dadu and Khanewal, India	678 (678) misoprostol, 720 (720) control	Age: 28 ± 5.7 Parity: n/a No education: 73%	Misoprostol (3/678) 0.4%, Control (*n* = 5/720) 0.6%	0.42 (0.08–2.18)
Misoprostol, TBAs						
Mobeen et al., 2010 [17]	RCT	46 villages in Chitral, Pakistan	533 (514) misoprostol, 583 (558) control	Age: 28 ± 5 Parity (3–5): 42.4% No education: 72.7%	Misoprostol 16.5% (85/514), Control 21.9% (122/558)	0.76 (0.59–0.97)
Prata et al., 2009 [32]	Quasi exp. trial	Rural villages of Tigray, Ethiopia	485 (485) misoprostol, 481 (481) control	Age: 27.7 ± 6.7 Parity: 3.4 ± 2.0 No education: 80.2%	Misoprostol 8.9% (43/485), Control 18.9% (91/481)	0.47 (0.33–0.66)
Walraven et al., 2005 [11]	RCT	26 villages of the North Bank East Health Division, The Gambia	629 (629) misoprostol + 4 placebo tablets, 599 (599) ergometrine^c^ + 3× placebo	Age: 25.9 ± 5.3 Parity(≥6): 18.6% No education: 95.2%	Misoprostol 11.0% (69/629), Ergometrine 12.0% (72/599)	0.91 (0.67–1.25)
Misoprostol, TBAs or self-administered						
Ejembi et al., 2014 [30]	Quasi exp. trial	5 communities in North-West Nigeria	1239 (1239) misoprostol, 231 (231) control	Age, parity, education: n/a	Misoprostol 8.1% (100/1239), Nothing 9.5% (22/231)	0.84 (0.54–1.30)
Prata et al., 2012a [33]	Quasi exp. trial	5 communities in North-West Nigeria	1421 (1421) misoprostol, 303 (303) control	Age, parity, education: n/a	Misoprostol 1.2% (17/1421) Nothing 7.6% (23/303)	0.16 (0.09–0.31)
Misoprostol, auxiliary midwives						
Chandhiok et al., 2005 [27]	Cluster-RCT	30 PHCs from 5 states in India	600 (600) misoprostol, 600 (600) standard (methergine^d^/ nothing)	Age:24.3 ± 3.6, Gravida (2–3): 56.3%, Literate: 62%	Misoprostol 0.7% (4/600), Control 0.8% (5/600)	0.80 (0.22–2.96)
Derman et al., 2006 [28]	RCT	4 PHCs of Belgaum district, India	812 (809) misoprostol, 808 (807) placebo	Age:23.3 ± 3.3 Parity (1–2): 58.1% Literacy: 62.9%	Misoprostol 6.4% (52/809), Control 12.0% (97/807)	0.53 (0.39–0.74)
Oxytocin^e^, auxiliary midwives						
Low et al., 2008 [34]	Quasi exp. trial	Public birth center in Morazan, Honduras	146 (146) oxytocin, 83 (83) control	Age:23.5 Parity (1–4): 49.2% Education: n/a	Oxytocin 12.3% (18/146), No oxytocin 19.3% (16/83)	0.64 (0.35–1.19)
Low et al., 2012 [35]	Quasi exp. trial	Public birth center in Morazan, Honduras	339 (339) intervention, 229 (229) pre-intervention	Age: 23.1 ± 6.5, Parity: 1.5 ± 2.1 Education: n/a	Intervention 5.9% (20/339), Pre-intervention 14.8% (34/229)	0.40 (0.23–0.67)
Misoprostol 2x200mcg, community health worker						
Nasreen et al., 2011 [36]	Quasi exp. trial	2 districts, northern Bangladesh	884 (884) misoprostol, 1008 (1008) control	Age:23.0 ± 4.8 Gravida: 2.6 ± 1.4 vs 2.1 ± 1.2 No education: 36.1% vs 21.2%	Misoprostol 1.6% (14/884), Control 6.4% (65/1008)	0.25 (0.14–0.43)
Oxytocin uniject device, community health worker						
Stanton et al., 2013 [29]	Cluster RCT	4 rural districts in Brong-Ahafo region, Ghana	689 (682) oxytocin 888 (888) control	Age: 27.5 ± 6.6. Parity(2–4): 43.3% No education: 48%	PPH-1:^f^ 2.6% (18/682) vs 5.5% (49/887) PPH-2: 3.8% (26/682) vs 10.8% (96/887) PPH-3: 4.1% (28/682) vs 11.1% (99/888) Severe PPH 0.1% (1/682) vs 0.9% (8/887)	PPH-1: 0.49 (0.27–0.88) PPH-2: 0.34 (0.18–0.63) PPH-3: 0.36 (0.20–0.66) Severe PPH: 1 (0.013–1.7)

*CCT* controlled cord traction, *CHW/O* community health worker/officer, *CI* confidence interval, *IU* international unit; mcg: microgram, *Ml* milliliters, *n/a* not available, *PHC* public health center, *PPH* postpartum hemorrhage, *RR* relative risk, *RCT* randomized controlled trial, *Quasi exp.* quasi experimental, *TBA* traditional birth attendant

^a^Population characteristics of intervention group if no statistical differences, reported in mean and standard deviation or percentage

^b^Tablets of misoprostol of 3x200micrograms if not otherwise described

^c^Ergometrine given as four tablets of 0.5 mg, as standard treatment at location of research

^d^Methergine given as intramuscular injections in a dose of 0.2 mg or 0.125 mg

^e^Oxytocin 10 international units, intramuscular injection

^f^PPH-1: blood loss ≥500 ml, PPH-2: PPH-1 plus any woman receiving early treatment for PPH, PPH-3: any woman without a quantitative blood loss measure who is referred to higher care for PPH

**Table 2:** Safety and acceptance of task shifting intervention in included studies (*n* = 12)

Study (first author, year of publication)	Study Design	Location	Population (age, parity, education)^a^	Training of intervention	Intervention – what/*who*^b^	Distribution complete - % of deliveries	Intervention – correct dose and/or timing (%)	Intervention – acceptance (%)
Diadhiou et al., 2011 [37]	Quasi-exp. trial	health center/post and maternity huts in 2 districts, Senegal	Age: 26.4 ± 5.5 Parity: 2.9 ± 2.2 No education: 43%	6 days course including 5 days on EoC and 1 day on misoprostol administration^c^	Misoprostol *auxiliary midwives*	16.1% (41/255) in Thies, 4.2% (9/214) in Kaolack, both percentages refer to distribution in maternity huts	Correct dose and timing: 100% (245/245) in HC/HP and MH	Recommended to friends: n/a Use at next delivery: n/a Willing to pay: 85.6% (160/187) in HC, 100% (8/8) in HP, 100% (50/50) in MH
Ejembi et al., 2014 [30]	Quasi- exp. trial	5 communities North-West Nigeria	Age, parity, education: n/a	TBAs: 6 days course on home-based EoC and misoprostol administration. Women: educated on misoprostol use at home visits of TBAs.	Misoprostol, distribution by drug keeper to *TBA, self-administration, friend/relative, doctor/midwife*	80.2% (1265/1577), total group *- TBA:*88.1% (919/1043) *- Self-administered:* 66.3% (106/160) *- Friend:* 67.1% (147/219) *- Midwife:*46.1% (47/102) - Attendant unknown for 53 deliveries	*- Total group:* dose 93.2% (1179/1265), time 87.3% (1104/1265) - *TBA:* dose 96.2% (884/919), time 88.9% (817/919) - *Self-administered:* dose 96.2% (102/106), time 77.4% (82/106) - *Friend:* dose 95.9% (141/147), time 85% (125/147) - *Midwife:* dose 93.6% (44/47), time 78.7% (37/47)	Recommended to friends: 99.7% (1260/1264) Use at next delivery: 99.4% (1256/1264) Willing to pay: 99.1% (1253/1264) Post-delivery data for this topic is missing for *n* = 1 delivery
Geller et al., 2014 [22]	Quasi- exp. trial	30 communities in rural Ghana	Age: 24.4 ± 6.56 Parity: 2.5 ± 2.05 Education: n/a	Training of midwives and CHWs on misoprostol administration. Use of pictorial charts for women.	Misoprostol, *self-administered*	65% (654/999) of misoprostol tablets distributed by midwives at antenatal care visits. *N* = 105 (96 at home, 9 institutional) women take misoprostol	Correct dose: n/a Correct timing: 98.9% (92/93) No data collected for institutional deliveries	Recommended to friends: 98.6% (71/72) Use at next delivery: 98.6% (71/72) Willing to pay: n/a Post-delivery data for this topic is missing for *n* = 21 deliveries
Mir et al.,2012 [31]	Quasi-exp trial	Dadu and Khanewal districts, India	Age 28 ± 5.7 Parity: n/a No education: 73%	Creating of community awareness & family education regarding use of misoprostol. 1 month before delivery women were assessed retention of knowledge. 15 days before delivery again briefed on information	Misoprostol, *self- administered*	88% (678/770) of women that delivered at home took misoprostol	Correct dose and timing: 95% (647/678)	Recommended to friends: 80% (616/770) Use at next delivery: 80% (616/770) Willing to pay: 74% (570/770)
Prata et al., 2012a [33]	Quasi-exp trial	5 rural communities North-West Nigeria.	Age, parity, education: n/a	TBAs were trained to counsel pregnant women about bleeding after delivery, the importance of delivery at a health facility, and the role of misoprostol and its administration.	Misoprostol, *TBA or self-administration*	79% (1421/1800) of women that were interviewed postpartum took misoprostol	Correct dose: 98% (1393/1421) Correct time: 88% (1250/1421)	Recommendations, use at next delivery, willing to pay: n/a
Prata et al., 2012b [38]	Quasi-exp trial	6 rural districts in Bangladesh	Age, parity, education: n/a	Training concerning various aspects of misoprostol and the usage of a delivery mat to measure blood loss.	Misoprostol, *RDRS trained and non-RDRS TBAs, self- administration, relative, nurse/doctor*	RDRS-trained TBA: 81.3% (1041/1280) Non-RDRS TBA: 69.9% (533/762) Relative 64.8% (250/386) Alone 67.7% (44/65) Doctor 7.6% (18/236) Nurse 67.7% (44/65)	Correct dose: n/a -*TBA (trained/untrained):* time 99.9% (1572/1574) - *other groups:* time 99.7% (355/356)	Recommended to friends: 98.6% (1903/1930) Use at next delivery: n/a Willing to pay: 84.6% (1633/1930) All women that were offered misoprostol, independent of birth attendant
Prata et al., 2014 [12]	Quasi-exp. trial	6 rural districts of Rangpur Division, Bangladesh	Age (15–29): 89.3% No education: 63% Gravidity (2+): 67.6%	RDRS trained TBAs received 2 days training on administration of misoprostol.	Misoprostol, *RDRS trained TBA, non-RDRS TBA, lay person*	67.4% (64.413/66489) RDRS trained TBA: 71% non-RDRS TBA 48% lay person 54%	Correct dose: n/a Correct time: n/a	Recommendations, use at next delivery, willing to pay: n/a
Rajbhandariet al., 2010 [39]	Quasi-exp. trial	30 clusters in 1 district in India, rural area.	Age: 25 yrs. Mean parity: 3; literate respondents: average 33%	Prenatal health education by female community health volunteers at home visits in 3–4 sessions. Other family members were involved. Advice on seeking prenatal care, planning institutional delivery, misoprostol, timely response to danger symptoms.	Misoprostol, *self-administered*	In study period (2006–2008) 74.5% (13969/18761) women took misoprostol. At end line of study 74% (604/816) of vaginal deliveries received misoprostol. 74% (447/604) of these women took misoprostol.	Correct dose: 98.2%(439/447) Correct time: 100% (447/447)	Recommendations, use at next delivery, willing to pay: n/a
Sanghvi et al., 2010 [10]	Quasi-exp. trial	8 districts in Afghanistan	Age: n/a Gravida > 1: 84.4% Education: n/a	Instructed by CHWs and SBAs during three home visits. Only received misoprostol when women were able to demonstrate understanding of the usage and risks.	Misoprostol, *self-administered*	99% (2021/2039) of pregnant women were offered misoprostol, 97.5% (1970/2021) accepted the drug. 70% (1421/2039) of all pregnant women, took the drug.	Correct dose: 99.8% (1418/1421) Correct time: 95.8% (1361/1421)	Recommended to friends: 92% (1876/2039) Use at next delivery: n/a Willing to pay: 88% (1794/2039)
Sibley et al., 2014 [5]	Quasi-exp. trial	3 districts in Amhara and Oromiya regions of Ethiopia	Age 20-34 yrs.: 76.5% No education: 71.9% Parity 2–4: 51%	Facility based Community Maternal and Neonatal Health Extension Program - training, no further specification.	Misoprostol, *health extension workers, CHWs, TBAs and self-administered*	58.9% (600/1019) of women received misoprostol. 97.5% (585/600) used it.	Correct dose and timing: 70.7% (412/585) Correct dose: 83.4% (483/585) Correct time: 84.1% (491/585)	Recommendations, use at next delivery, willing to pay: n/a
Smith et al., 2014a [13]	Quasi-exp. trial	2 districts in Grand Bassa county, Liberia	Age, parity, education: n/a	Explanation of misoprostol use to women at antenatal care visits or at home visits by district reproductive health supervisor.	Misoprostol, *self-administered*	53.7% (980/1826) of all women received misoprostol. Of a sample of 550 women who received misoprostol 302 delivered at home, 87.7% (265/302) of them took the drug.	Correct dose: n/a Correct time: 63% (167/265)	Recommended to friends: 99.6% (258/259) Use at next delivery: 98.1% (254/259) Willing to pay: 54.6% (142/260) For some questionnaires data were missing. Data shown are independent of distributor of misoprostol
Smith et al., 2014b [40]	Quasi-exp. trial	Mundri East County and South Sudan, Sudan	Age, parity, education: n/a	Education of misoprostol use to women at pre-natal care visits or home visits by SBAs and maternal health workers	Misoprostol, *self-administered*	84.9% (787/927) of women received misoprostol during pregnancy. 98.9% (527/533) of women delivering at home received misoprostol. Postpartum data were gathered for 76.1% (599/787) of women of whom 81% (485/599) had home delivery with misoprostol.	Correct dose: n/a Correct time: 98.6% (478/485)	Recommended to friends: 95.1% (461/485) Use at next delivery: 99.0% (480/485) Willing to pay: 99.0% (480/485)

Articles sorted on alphabetical order. *n/a* not available, *CHW* community health worker, *EoC* emergency obstetric care, *HC* health centre, *HP* health post, *MH* maternity hut, *TBA* traditional birth attendant, *RDRS* Rangpur Dinajpur Rural Services, *SBA* skilled birth attendant

^a^Population characteristics of only intervention group if no statistical differences

^b^tablets of 3x200micrograms if not otherwise described

^c^misoprostol administration refers to misoprostol use as prevention for PPH and focuses on correct storage, dose, time and route of misoprostol administration, as well as side effect management

All studies assessed the administration of uterotonics; misoprostol tablets or oxytocin injections. Misoprostol tablets for self-administration were distributed at antenatal (home) visits (*n* = 5), at delivery (*n* = 12) or both (*n* = 4). Controlled cord traction, uterine massage and uterine tone assessment and their effect on reduction of PPH were not reported in any of the studies. Therefore the main focus of the remainder of this article will be on task shifting of uterotonic admission.

### Risk of bias within studies

**Fig. 2:**
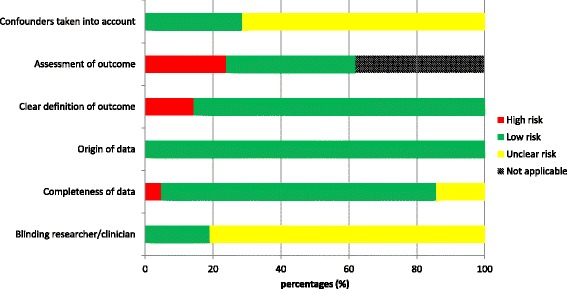
Bar graph showing the risk distribution according to the different variables on which 21 articles were assessed. Blinding of researcher/clinician was only evaluated if intervention was compared to control group

The overall risk assessment is summarized in Fig. 2, the individual study risk of bias assessment is available as a Additional file 2.

#### Detection bias

In 14% (*n* = 3) of 21 studies both delivered women as participants were blinded for intervention and outcome. In one study the outcome was measured by workers who were not involved in the distribution process. In the remaining studies participants performing the intervention were also involved in the measurement of outcomes. In none of the articles a bias effect is mentioned and because of possible influence on outcomes, risk of bias was scored as unclear.

#### Attrition bias

Data on baseline characteristics were obtained at antenatal care visits in facility centers or during home visits. Data on intervention and outcome measures were obtained at delivery by auxiliary midwives, community health workers or traditional birth attendants by interviewing women postpartum. All health workers received training on how to collect data and therefore low risk of bias for origin of data was scored. In 81% (*n* = 17) of studies were less than 10% missing data of outcome measures or loss to follow up and therefore low risk of bias on completeness of data was scored. One article had more than 10% missing data [22].

#### Reporting bias

In 86% (*n* = 18) of articles the definition of PPH was clear. In the other articles PPH was defined as ‘need for referral’ or ‘heavy bleeding’. Blood loss was objectively measured in 62% (*n* = 8) of studies with a calibrated scale. In 38% (*n* = 5) of studies blood loss was visually estimated by health workers or blood loss was subjectively estimated by delivered women and was therefore scored as a high risk of bias. PPH was not measured in eight articles and scored as ‘not applicable’.

#### Possible confounders

Confounders were scored as low risk of bias in randomized controlled trials without differences in baseline characteristics. In 71% (*n* = 15) of studies possible confounders were not reported and therefore risk of bias was scored ‘unclear’.

### Results of individual studies

#### Primary outcome: PPH incidence

In thirteen studies (15.197 women), the primary outcome assessed was the incidence of PPH when uterotonics were provided by unskilled birth attendants.

The incidence of PPH in delivered women who received misoprostol tablets (*n* = 10) was compared to the incidence in women treated by the standard care of no uterotonics (*n* = 8), ergometrine (*n* = 1) or methergine (*n* = 1). Intramuscular oxytocin injections (10 international units) were provided in three studies, in one of these a Uniject device, a disposable automatic syringe, was used. Oxytocin was administered by auxiliary midwives or community health workers. The relative risks of PPH incidence varied from 0.16 to 1 in favor of task shifting. For seven of thirteen articles relative risks were statistically significant [17, 28, 29, 32, 33, 35, 36].

In all studies, educational programs were organized before the start of the interventional trial. Most educational programs for community health workers, auxiliary midwives or traditional birth attendants included a multiple-day course on aspects of AMTSL, mainly focusing on administration of uterotonics. Pregnant women were educated on self-administration by nurses or health workers at antenatal care visits, both at home or in a clinic.

#### Secondary outcome: Acceptance and safety of task shifting

In twelve studies [5, 10, 12, 13, 22, 30, 31, 33, 37–40] the primary outcome assessed was women’s acceptance and safety of community distribution of uterotonics [Table 2]. In all studies administration of uterotonics was evaluated.

#### Women’s acceptance

Of delivered women who self-administered misoprostol tablets postpartum or received tablets from traditional birth attendants, 80% to 99.7% (7 studies, 6445 women) recommended taking misoprostol tablets at delivery to family or friends. Approximately the same percentage, 80% to 99.4% (5 studies, 2677 women), would use misoprostol at their next delivery and 54.6% to 100% (7 studies, 6090 women) were willing to pay for the tablets. Of delivered women who received tablets from auxiliary midwives, 85.6% to 100% (1 study, 218 women) were willing to pay for tablets. No data were available for women who received tablets from community health workers.

#### Safety of intervention

Of delivered women who self-administered misoprostol tablets or received tablets from traditional birth attendants 83.4% to 99.8% (5 studies, 4719 women) took misoprostol tablets at the correct dose and 63% to 100% (9 studies, 6757 women) took tablets at the correct time, after delivery of the baby and before placental delivery. Both correct dose and time was mentioned in two articles and achieved by 70.7% to 95% of women (*n* = 1304). Approximately the same percentages were observed for auxiliary midwives and community health workers, 83.4% (1 study, 483 women) took the correct dose, 84.1% (1 study, 491 women) at the correct time. Both correct dose and time was mentioned in two articles and achieved by 70.7% to 100% of women (*n* = 657). Incorrect use of misoprostol mostly included taking a lower dose or waiting too long for taking the tablets. Eight articles explicitly described that misoprostol was not taken before delivery of the baby, in one article less than 2 % of the pregnant women took misoprostol pre-infant delivery.

#### Adverse effects of uterotonics

Adverse effects of misoprostol or oxytocin in the community setting were mentioned in fourteen articles [10,11,13,17,22,27,28,30,31,32,36,37,39,40, Additional file 3]. Mild-to-moderate adverse effects of misoprostol included nausea, vomiting, shivering and/or fever. There were no significant differences in these adverse effects between intervention and control groups of no uterotonics, ergometrine (*n* = 1 study) or methergine (*n* = 1 study).

## Discussion

This systematic review about task shifting within AMTSL interventions shows that only administration of uterotonics has been evaluated in community and health facility settings in low- and middle-income countries. No data are available for task shifting of controlled cord traction, uterine massage or uterine tone assessment and its effect on incidence of PPH.

Compared to no AMTSL, the administration of uterotonics by traditional birth attendants, auxiliary midwives, community health workers or the woman herself reduced the incidence of PPH. Task shifting of this part of AMTSL to unskilled birth attendants was generally accepted by women and care providers and was reported to be a safe intervention.

Community distribution of misoprostol and oxytocin by auxiliary midwives and lay health workers as a strategy to increase uterotonic coverage is currently included in WHO guidelines on the prevention of postpartum hemorrhage [4]. The results of this review confirm the safety and effectiveness of this strategy. Administration of misoprostol or oxytocin self-administered by delivered women is not yet recommended in WHO guidelines, and could be considered a strategy to further increase access to AMTSL.

A number of challenges in improving the implementation of AMTSL through task shifting have been suggested. First, misoprostol can be used as an abortifacient and mistimed administration could lead to premature deliveries [39]. Although misoprostol is recognized as an essential medicine by the WHO, worries of mistimed administration have prevented the registration in many countries [41]. While no data were available for number of abortions or preterm deliveries, this remains a potential serious side effect of mistimed administration of misoprostol. The results of this study show that the vast majority of women took or received tablets at the correct time and in the correct dose. In addition, programs that encourage return of misoprostol tablets in case it was not used (e.g. in facility delivery where oxytocin was available), had a near-complete recovery rate [22]. Our results are in line with a systematic review of Smith et al. [42], who described strategies and their safety when distributing misoprostol in the community. They identified 18 studies and showed that 0.06% (*n* = 7) of 12.615 women incorrectly used misoprostol. No significant differences were seen for the number of stillbirths or neonatal deaths in case uterotonics were provided.

Secondly, an emphasis on task shifting elements of AMTSL could send a mixed-message and may inadvertently stimulate pregnant women to deliver at home without skilled birth attendants. Therefore, the message should always be to promote facility based delivery and only use pragmatic rescue treatment if other options fail. As such, task shifting could be considered complementary in creating community awareness as an intermediate solution towards universal facility based deliveries. In fact, introducing misoprostol distribution programs and associated education training programs for implementation have been reported to increase awareness of the importance of medical care and referrals to Emergency Obstetric Care facilities [7, 31]. Prata et al. [38] observed that level of training is directly proportional to distribution rates of uterotonics, emphasizing the importance of education in creating community awareness. An additional advantage of an intense training program with task shifting of AMTSL to community health workers and traditional birth attendants may be to increase their awareness about referral indications and provide a bridge between community-based traditional care and facility-based obstetric care [7]. Task shifting of AMTSL may also provide an opportunity for facility-based obstetric care to improve quality of care in light of the continuing shortage of skilled birth attendants and to reduce workload for midwives. For example, a randomized controlled trial in a tertiary hospital in Ghana (clinicaltrials.gov, registration number: NCT02223806; [43]*)* suggested that the final step of postpartum assessment of the uterine tonus can be effectively conducted by patients, whilst regularly monitored by midwives (unpublished data). Recent evidence indicates that omission of CCT has little effect on the risk of severe hemorrhage and the WHO recommends not to include CCT in hemorrhage prevention programs for non-hospital settings [8, 9].

Strengths of this review include the broad focus to include multiple components of AMTSL. In contrast to earlier reviews, we did not only focus on the effect on PPH incidence and safety, but also included women’s acceptance of the interventions [20, 42]. Limitations include our inability to conduct a meta-analysis due to heterogeneity of interventions, outcome measurements and contexts. Data were collected at antenatal care visits, during delivery or a few days or weeks postpartum by health workers or traditional birth attendants. Postpartum hemorrhage was measured using different methods varying from visual estimation to using a calibrated scale, possibly explaining the high differences in incidence of PPH between the included studies. Additional file 2 provides more detailed information on measurements of postpartum hemorrhage among the different studies. Educational programs differed between studies, varying from 1-day programs to 7-day programs which is likely to influence the level of skills. An additional limitation is that we only included peer-reviewed literature, which may have excluded intervention reports from the grey literature, for example from non-governmental organizations working in LMIC. All studies evaluating the safety of community distribution of misoprostol, compared reduction of PPH to standard care, which is (mostly) no use of uterotonics. No trials exist comparing the effect on PPH of uterotonics by unskilled birth attendants compared to administration by skilled attendants. However, by not only focusing on the effect of uterotonic administration itself but by also evaluating the effect and safety of task shifting in rural areas, this systematic review has also included safety and acceptance of community distribution.

This review supports the growing body of evidence that the potential of human resources should be maximized at all levels of the health-care system to reduce the burden of maternal morbidity and mortality. In line with this the Senegal Ministry of Health agreed in their national strategic plan for 2014–18 that misoprostol could be provided by auxiliary midwives in maternity huts [44]. In the prevention of PPH as major contributor to maternal mortality, the efficacy and safety of task shifting of all elements (except for controlled cord traction, which is discouraged by the WHO in case skilled birth attendants are absent) within AMSTL should be evaluated in future studies.

## Conclusion

Task shifting of (parts of) AMSTL to community health workers, traditional birth attendants or self-administration has been explored in response to the shortages in skilled birth attendants and facility-based deliveries in low- and middle income countries. This systematic review showed that so far only task shifting in uterotonics (oxytocin and misoprostol) administration has been reported. The administration of misoprostol tablets by CHWs, traditional birth attendants or self-administered proved to be effective, safe and accepted in low resource settings. The administration of intramuscular oxytocin by CHWs and auxiliary midwives proved to be effective in reducing PPH. Educational programs are the key element for improvement of implementation of AMTSL and creating awareness of importance of timely referral for emergency obstetric care. As a comprehensive PPH-reduction-strategy based on the AMTSL requires implementation of all steps (except for controlled cord traction, which is discouraged by the WHO in case skilled birth attendants are absent) expanding task shifting to other relevant components of AMTSL will provide additional opportunities to prevent maternal morbidity and mortality due to PPH.

## Additional files

Additional file 1: Full search strategy and list of synonyms. (DOCX 19 kb)

Additional file 2: Individual study risk of bias assessment, according to quality assessment tool. (XLSX 16 kb)

Additional file 3: Side effects of misoprostol during pregnancy / delivery (*n* = 14). (DOCX 17 kb)

## Abbreviations

AMTSL: Active management of the third stage of labor
CCT: Controlled cord traction
CHW: Community health worker
CI: Confidence interval
EOC: Emergency obstetric care
FIGO: International federation of gynecology and obstetrics
ICM: International confederation of midwives
LMIC: Low- and middle income country
Ml: Milliliters
PPH: Postpartum hemorrhage
RR: Relative risk
SBA: Skilled birth attendant
TBA: Traditional birth attendant
WHO: World Health Organization
