# Supplementary material for: Task shifting in active management of the third stage of labor: a systematic review
Source: BMC Pregnancy Childbirth. 2018 Feb 6;18:47. doi: 10.1186/s12884-018-1677-5 (PMC5801808; doi:10.1186/s12884-018-1677-5)
Supplement: Supplementary file 1 — Full search strategy and list of synonyms. (DOCX 19 kb) [file 12884_2018_1677_MOESM1_ESM.docx]

**Supplementary file 1. Exact search query**

| **September 2^nd^, 2015. PubMed search syntax** | | |
| --- | --- | --- |
|  |  | Hits |
| Domain #1 | ((massage[MeSH Terms]) OR uterine inertia[MeSH Terms]) OR umbilical cord[no:exp]) OR uterine muscle[MeSH Terms]) OR oxytocics[MeSH Terms]) OR misoprostol[MeSH Terms]) OR labor stage, third[MeSH Terms])) OR ((amtsl[Title/Abstract]) OR third stage of labor[Title/Abstract]) OR third stage of labour[Title/Abstract]) OR third stage labor[Title/Abstract]) OR third stage labour[Title/Abstract])) OR ((uterotonic[Title/Abstract]) OR uterotonics[Title/Abstract]) OR misoprostol[Title/Abstract]) OR oxytocin[Title/Abstract]) OR oxytocine[Title/Abstract]) OR syntocinon[Title/Abstract]) OR cytotec[Title/Abstract]) OR oxytocic[Title/Abstract]) OR oxytocics[Title/Abstract]) OR ocytocin[Title/Abstract]) OR pitoci[Title/Abstract])) OR ((cord clamping[Title/Abstract]) OR cord traction[Title/Abstract])) OR ((uterine tone[Title/Abstract]) OR uterine tonus[Title/Abstract]) OR uterine contraction[Title/Abstract]) OR uterus contraction[Title/Abstract])) OR uterine massage[Title/Abstract])) OR ((abdominal massage[Title/Abstract]) OR fundal massage[Title/Abstract]) | 75.115 |
| Determinant #2 | ((delivery of healthcare[MeSH Terms]) OR self administration[MeSH Terms]) OR health manpower[MeSH Terms]) OR community health workers[MeSH Terms]) OR midwifery[MeSH Terms])) OR ((task shifting[Title/Abstract]) OR shifting task[Title/Abstract]) OR task shift[Title/Abstract]) OR shift task[Title/Abstract]) OR skill mix[Title/Abstract]) OR skills mix[Title/Abstract]) OR substitution of physicians[Title/Abstract]) OR nurse substitution[Title/Abstract]) OR task delegation[Title/Abstract]) OR task sharing[Title/Abstract]) OR skill substitution[Title/Abstract]) OR personnel mix[Title/Abstract]) OR self administration[Title/Abstract])) OR ((((birth attendants[Title/Abstract]) OR health workers[Title/Abstract]) OR self administered[Title/Abstract]) OR shifting tasks[Title/Abstract])) OR (community based[Title/Abstract]) OR (community distribution[Title/Abstract]) OR (self assessment[Title/Abstract]) OR (self-assessment[Title/Abstract]) OR (self assessed[Title/Abstract]) OR (self-assessed[Title/Abstract]) | 915.571 |
| Results | #1 AND #2 | 1972 |

| **September 4^nd^, 2015. EMBASE search syntax** | |  |
| --- | --- | --- |
|  | | Hits |
| Domain #1 | ‘amtsl’:ab,ti OR ‘third stage of labor’:ab,ti OR ‘third stage of labour’:ab,ti OR ‘third stage labor’:ab,ti OR ‘third stage labour’:ab,ti OR ‘uterotonic’:ab,ti OR ‘uterotonics’:ab,ti OR ‘misoprostol’:ab,ti OR ‘oxytocin’:ab,ti OR ‘oxytocine’:ab,ti OR ‘syntocinon’:ab,ti OR ‘cytotec’:ab,ti OR ‘oxytocic’:ab,ti OR ‘oxytocics’:ab,ti OR ‘ocytocin’:ab,ti OR ‘pitocin’:ab,ti OR ‘cord clamping’:ab,ti OR ‘cord traction’:ab,ti OR ‘uterine tone’:ab,ti OR ‘uterine tonus’:ab,ti OR ‘uterine contraction’:ab,ti OR ‘uterus contraction’:ab,ti OR ‘uterine massage’:ab,ti OR ‘abdominal massage’:ab,ti OR ‘fundal massage’:ab,ti OR ‘atony’:ab,ti OR ‘uterus tonus’:ab,ti OR ‘uterus tone’:ab,ti OR ‘uterus massage’:ab,ti OR ‘abdomen massage’:ab,ti OR ‘fundus massage’:ab,ti OR 'massage'/exp OR ‘uterine atony’/exp OR ‘umbilical cord’/mj OR ‘myometrium’/exp OR ‘oxytocic agent’/exp OR ‘misoprostol’/exp OR ‘labor stage 3’/exp | 62.471 |
| Determinant #2 | ‘task shifting’:ab,ti OR ‘shifting task’:ab,ti OR ‘task shift’:ab,ti OR ‘shift task’:ab,ti OR ‘skill mix’:ab,ti OR ‘skills mix’:ab,ti OR ‘substitution of physicians’:ab,ti OR ‘nurse substitution’:ab,ti OR ‘task delegation’:ab,ti OR ‘task sharing’:ab,ti OR ‘skill substitution’:ab,ti OR ‘personnel mix’:ab,ti OR ‘self administration’:ab,ti OR ‘birth attendants’:ab,ti OR ‘health workers’:ab,ti OR ‘self administered’:ab,ti OR ‘shifting tasks’:ab,ti OR 'health care delivery'/mj OR 'drug self administration'/exp OR 'health care manpower'/exp OR 'health auxiliary'/exp OR 'nurse midwifery'/exp OR ‘community based’:ab,ti OR ‘community distribution’:ab,ti OR ‘self assessment’:ab,ti OR ‘self assessed’:ab,ti | 174.791 |
| Results | #1 AND #2 | 633 |

| **September 6^nd^, 2015. The Global Health Library search syntax** | |  |
| --- | --- | --- |
|  | | Hits |
| Domain #1 | ("amtsl" OR "uterotonic" OR "uterotonics" OR "misoprostol" OR "oxytocin" OR "oxytocine" OR "syntocinon" OR "cytotec" OR "oxytocic" OR "oxytocics" OR "ocytocin" OR "pitocin" OR "uterine contraction" OR "massage" OR "uterine inertia" OR "umbilical cord" OR "uterine muscle" OR "myometrium") | 60.159 |
| Determinant #2 | ("self administration" OR "self-assessed" OR "self assessment" OR "self-assessment" OR "health care delivery" OR "health manpower" OR "community health workers" OR "midwifery") | 136.267 |
| Results | #1 AND #2 | 561 |

| **September 8^nd^, 2015. Cochrane Library search syntax** | | |
| --- | --- | --- |
|  |  | Hits |
| Domain #1 | (‘amtsl’ or ‘third stage of labor’ or ‘third stage of labour’ or ‘third stage labor’ or ‘third stage labour’ or ‘uterotonic’ or ‘uterotonics’ or ‘misoprostol’ or ‘oxytocin’ or ‘oxytocine’ or ‘syntocinon’ or ‘cytotec’ or ‘oxytocic’ or ‘oxytocics’ or ‘ocytocin’ or ‘pitocin’ or ‘cord clamping’ or ‘cord traction’ or ‘uterine tone’ or ‘uterine tonus’ or ‘uterus tone’ or ‘uterus tonus’ or ‘uterine contraction’ or ‘uterus contraction’ or ‘uterine massage’ or ‘uterus massage’ or ‘abdomen massage’ or ‘abdominal massage’ or ‘fundus massage’ or ‘fundal massage’ or ‘atony’):ti,ab or [labor stage, third] or [uterine inertia] or [oxytocics] | 5.169 |
| Determinant #2 | (‘task shifting’ or ‘shifting task’ or ‘skill mix’ or ‘skills mix’ or ‘substitution of physicians’ or ‘nurse substitution’ or ‘task delegation’ or ‘task sharing’ or ‘skill substitution’ or ‘personnel mix’ or ‘self administration’ or ‘birth attendants’ or ‘health workers’ or ‘self administered’ or 'community based' or 'community distribution' or ‘self-assessment’ or ‘self assessment’ or ‘self-assessed’ or ‘self assessed’):ti,ab or [self administration] or [health manpower] or [health service accessibility] or [community health workers] | 35.194 |
| Results | #1 AND #2 | 185 |

| **September 10^nd^, 2015. POPLINE search syntax** | | |
| --- | --- | --- |
|  |  | Hits |
| Domain #1 | “amtsl” OR “third stage labor” OR “third stage labour” OR “3rd stage labour” OR “3rd stage labor” OR “final stage labour” OR “final stage labor” OR “third stage childbirth” OR “3rd stage childbirth” OR “final stage childbirth” OR “third stage delivery” OR “3rd stage delivery” OR “final stage delivery” OR “uterotonic” OR “uterotonics” OR “misoprostol” OR “oxytoc*” OR “syntocinon” OR “cytotec” OR “ocytoc*” OR “pitocin*” OR “cord tract*” OR “umbilical tract*” OR “uterine ton*” OR “uterus ton*” OR “uterine contract*” OR “uterus contract*” OR “uterine massag*” OR “uterus massage*” OR “abdominal massag*” OR “fundal massag*” OR “uterine rub*” OR “uterus rub*” OR “atony” OR “uterine inertia” | 11.896 |
| Determinant #2 | "task shifting" OR "shifting task" OR "task shift" OR "shift task" OR "skill mix" OR "skills mix" OR "substitution of physicians" OR "nurse substitution" OR "task delegation" OR "task sharing" OR "skill substitution" OR "personnel mix" OR "self administration" OR "selfadministration" OR "birth attendants" OR "health workers" OR "self administered" OR "selfadministered" OR "shifting tasks" OR "community based" OR "community distribution" OR "self assessment" OR "selfassessment" OR "self assessed" OR "selfassessed" OR “health manpower” OR “community health workers” | 18.323 |
| Results | #1 AND #2 | 671 |
