# Supplementary material for: Task shifting in active management of the third stage of labor: a systematic review
Source: BMC Pregnancy Childbirth. 2018 Feb 6;18:47. doi: 10.1186/s12884-018-1677-5 (PMC5801808; doi:10.1186/s12884-018-1677-5)
Supplement: Supplementary file 3 — Side effects of misoprostol during pregnancy / delivery (n = 14). (DOCX 17 kb) [file 12884_2018_1677_MOESM3_ESM.docx]

**Table 3. Side effects of misoprostol during pregnancy / delivery (n=14) in community settings in LMICs.**

| **Study (first author, year of publication)** | **Side effects (nausea, vomiting, fever, shivering)*** |
| --- | --- |
| Chandhiok et al., 2005 [27] | *Misoprostol(n=600) vs control; methergine** (n=589)/nothing(n=11)*  -Nausea 10.2% (61/600) vs 20.2% (121/600) p<0.001  -Vomiting 5% (30/600) vs 5.7% (34/600)  -Temp > 38 9.7% (58/600) vs 4.3% (26/600) p<0.001  -Shivering (mild or severe) 48.7% (292/600) vs 19.2% (115/600) p<0.001 |
| Diadhiou et al., 2011 [37] | *Misoprostol (n=245)*  -Nausea 0.4% (1/245)  -Vomiting 0.4% (1/245)  -Fever 3.7% (9/245)  -Shivering 32.2% (79/245) |
| Derman et al., 2006 [28] | *Misoprostol (n=809) vs control (n=807)*  -Fever 4.2% (34/809) vs 1.1% (9/807)  -Shivering 52.2% (419/803) vs 17.3% (140/807)  There were no differences in the rates of nausea or vomiting |
| Ejembi et al., 2014 [30] | *Misoprostol (n=1264) vs control (n=313) ; home- and facility delivery*  -Fever 12% (150/1250)  -Shivering 43% (539/1253)  No side effects mentioned for control group. Numbers differ depending number of respondents. |
| Geller et al., 2014 [22] | *Misoprostol (n= 102) vs control (n=107)*  -Fever 4.3% (4/93)  -Shivering 29% (27/93)  -Other (not specified) 2.2% (2/93)  No side effects mentioned for control group |
| Mir et al., 2012 [31] | *Misoprostol (n=678) vs control (n=*720)  -Nausea 6.6% (45/678)  -Vomiting 3.8% (26/678)  -Fever 11.2% (76/678)  -Shivering 28.2% (191/678)  No side effects mentioned for control group |
| Mobeen et al., 2010 [17] | *Misoprostol(n=533) vs placebo(n=583)*  -Nausea 1.5% (8/533) vs 1.4% (8/583)  -Vomiting 0.6% (3/533) vs 0.5% (3/583)  -Fever 0.8% (4/533) vs 1.2% (7/583)  -Shivering 9.4% (50/533) vs 3.9% (23/583) p<0.0001 |
| Nasreen et al., 2011 [36] | *Misoprostol (n=884) vs control (n=1008)*  - 1.2% (11/884) had transient increase in shivering, abdominal pain, fever, oedema |
| Prata et al., 2009 [32] | *Misoprostol (n=485) vs control (n=481)*  -Vomiting 6.6% (32/485) vs 4.8% (23/481)  -Shivering 12.2% (59/485) vs 6.7% (32/481) OR 1.94 (1.23-3.05) |
| Rajbhandari et al., 2010 [39] | *Misoprostol(n=444) vs non-users(n=372)*  -Nausea 3.4% (15/444) vs 6.2 (23/372) OR 0.51 (0.29-0.89)  -Vomiting n/a  -Fever 9.0% (40/444) vs 9.4% (35/372)  -Shivering 18.5% (82/444) vs 15% (56/372) |
| Sanghvi et al., 2010 [10] | *Intervention(n=2039) vs comparison (n=1148)*  -Nausea 7.0% (143/2039) vs 20.0% (230/1148) p<0.001  -Vomiting 3.9% (80/2039) vs 9.5% (109/1148) p<0.001  -Fever 8.5% (173/2039) vs 32.6% (374/1148) p<0.001  -Shivering 28.2% (575/2039) vs 33.2% (381/1148) p<0.001 |
| Smith et al., 2014-A [13] | *Misoprostol (n=265)*  -At least one side effect (nausea, vomiting, fever or shivering) 54% (143/265) |
| Smith et al., 2014-B [40] | *Misoprostol at home delivery (n=527)*  -At least one side effect (nausea, vomiting, fever or shivering) 100% (527/527) |
| Walraven et al., 2005 [11] | *Misoprostol(N=630) vs Ergometrine***(N=599)*  -Nausea 0.9% (6/630) vs 2.3% (14/599)  -Vomiting 2.9% (18/630) vs 5.7% (34/599) RR 0.50 (0.29-0.88)  -Fever n/a  -Shivering 32.1% (202/630) vs 11.7% (70/599) RR 2.74 (2.14-3.52) |

Articles sorted on alphabetical order. LMICs: low- and middle-income countries; n/a: not available; RR: relative risk; OR odds ratio

*p value, odds ratio or relative risk noted if significant difference

**Methergine given as intramuscular injections in a dose of 0.2 milligrams or 0.125 milligrams

***Ergometrine given as four tablets of 0.5 milligrams, as standard treatment at location of research
